# Fibrinogen storage disease without hypofibrinogenemia associated with estrogen therapy

**DOI:** 10.1186/1471-230X-5-36

**Published:** 2005-11-15

**Authors:** Z Simsek, O Ekinci, M Cindoruk, T Karakan, B Degertekin, G Akyol, S Unal

**Affiliations:** 1 Gazi University Faculty of Medicine, Gastroenterology Department, Ankara, Turkey; 2 Gazi University Faculty of Medicine, Pathology Department, Ankara, Turkey

## Abstract

**Background:**

Cytoplasmic inclusion bodies within hepatocytes may have different etiologies, including the Endoplasmic Reticulum Storage Diseases (ERSDs). ERSD is a pathological condition characterized by abnormal accumulation of proteins destined for secretion in the endoplasmic reticulum of hepatocytes; it may be congenital (primary) or acquired (secondary). Fibrinogen storage disease is a form of ERSD.

**Case Presentation:**

We present a case of fibrinogen storage disease secondary to estrogen replacement therapy. Its causal relationship to the drug is shown by histological, immunohistochemical and ultrastructural studies of paired liver biopsies obtained during and after the drug therapy.

**Conclusion:**

The liver biopsies of patients with idiopathic liver enzyme abnormalities should be carefully evaluated for cytoplasmic inclusion bodies and, although rare, fibrinogen deposits.

## Background

Cytoplasmic inclusions in hepatocytes are encountered in a wide range of clinical conditions including chronic hepatitis B, α_1_-antitrypsin deficiency, type IV glycogenosis, Lafora's disease, fibrinogen storage disease, and in drug reactions [1,2]. Endoplasmic Reticulum Storage Disease (ERSD) of the liver is a pathological condition characterized by abnormal accumulation of proteins destined for secretion in the endoplasmic reticulum. The term ERSD encompasses α_1_-antitrypsin deficiency, the prototype disorder, plus fibrinogen storage disease and α_1_-antichymotrypsin deficiency [3]. ERSD can be congenital or acquired. The congenital form is associated with the accumulation of only one protein, due to its altered structure. The accumulation is permanent and affects mainly the rough endoplasmic reticulum (RER). The most common cause of this condition is α1-antitrypsin deficiency. Acquired [secondary] form occurs due to exogenous agents or in the presence of concomitant diseases such as infections, and therefore structurally normal proteins accumulate in several cellular organelles.

In this article, we report a case of fibrinogen storage disease of the liver secondary to the use of oral contraceptives. This is the first reported case of fibrinogen storage disease secondary to estrogen ingestion. Informed consent was obtained from the patient regarding her recruitment in this case study.

## Case presentation

A forty-year-old female was referred to our department for evaluation of elevated liver enzymes in May, 2004. She had no systemic symptoms. A year before, she had had a total abdominal hysterectomy and bilateral salpingo-oophorectomy. Since then, she had been on hormone replacement therapy with estrogen. Six months ago, during her routine follow-up, elevation of transaminase levels was noted. This elevation persisted for the next 6 months. She was then referred to our department for further investigation.

Laboratory features of the patient regarding this period are shown in Table 1. Detailed questioning of the patient revealed no history of past alcohol abuse. Screening for viral antigens and for antibodies against them, as well as screening for viral nucleotide-acid sequences (including HCV-RNA/-cDNA PCR), found no evidence of infection. Levels of auto-antibodies, serum transferrin saturation, serum ferritin, serum ceruloplasmin, serum copper, serum α1-antitrypsin, and 24-hour urinary copper excretion were either negative or within normal limits. Abdominal ultrasonography was unremarkable. Doppler ultrasound imaging of portal and hepatic veins and the hepatic artery revealed no thrombosis.

**Figure 1:**
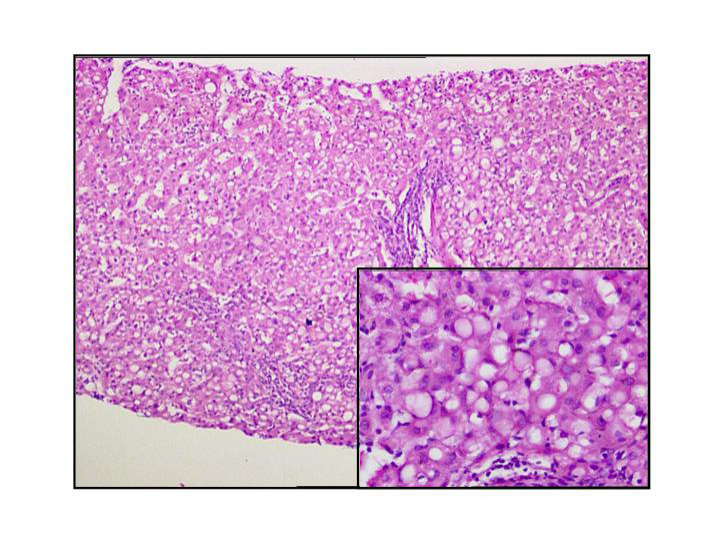
Intracytoplasmic inclusions with ground-glass appearance affecting most of the hepatocytes in this area. Mononuclear inflammatory infiltrate in a portal area is also seen. Hematoxylin-eosin, ×40. Inset: Higher magnification of this area, (H&E, ×200).

**Figure 2:**
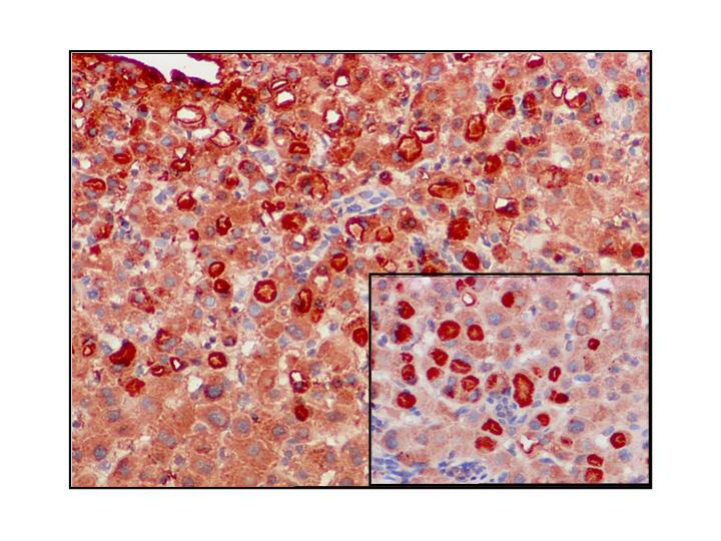
Strong and diffuse immunohistochemical positivity of the inclusions for fibrinogen, ×40. İnset: Same area with higher magnification. (×200).

A liver biopsy was performed. Histopathological examination revealed focal necrosis and mild fibrotic activity. Macrovesicular steatosis affecting 5% of hepatocytes, in addition to several foci of prominent nuclear pleomorphism and hyperchromasia were also noted. However, the most remarkable finding was the presence of abundant intracytoplasmic, sharply bordered, pale eosinophilic inclusions that exhibited a ground-glass appearance (Figure 1). Periodic acid-Schiff (PAS), diastase-treated PAS and colloidal iron stains were applied. Immunohistochemistry for hepatitis B surface (mouse monoclonal antibody clone: Tg, Neomarkers) and core (mouse monoclonal antibody clone: Tordji-22, Signet) antigens, α1-antitrypsin (rabbit antibody, Zymed), fibrinogen (rabbit anti-human antibody, Dako), α fetoprotein (rabbit polyclonal antibody, Signet), complement 3 (mouse monoclonal antibody clone: HAV 3–4, Dako) and complement 4 (rabbit anti-human antibody, Dako) were performed. A three-step streptavidin biotin method using AEC as chromogen was applied in the immunohistochemical procedure. The inclusions were negative to PAS and colloidal iron. No reactivity with antibodies against hepatitis antigens, α1-antitrypsin and complement 3 was observed. Diffuse and strong positivity was observed for fibrinogen in cytoplasmic inclusions (Figure 2). We also detected less positivity for complement 4 and α-fetoprotein.

**Figure 3:**
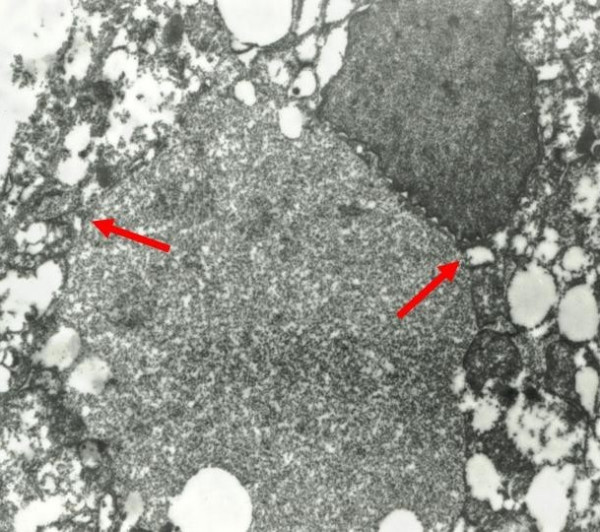
On ultrastructure, inclusions appear as finely granular, homogeneous material bounded by membranes (arrows). ×4400

Paraffin-embedded tissue was processed for electron microscopic examination. Ultrastructurally, the inclusions were found to be moderately electron-dense, finely granular, homogenous bodies that are encircled by membranes, corresponding to a dilated endoplasmic reticulum (Figure 3). There were no parallel arrays of tubular structures in the inclusions.

**Figure 4:**
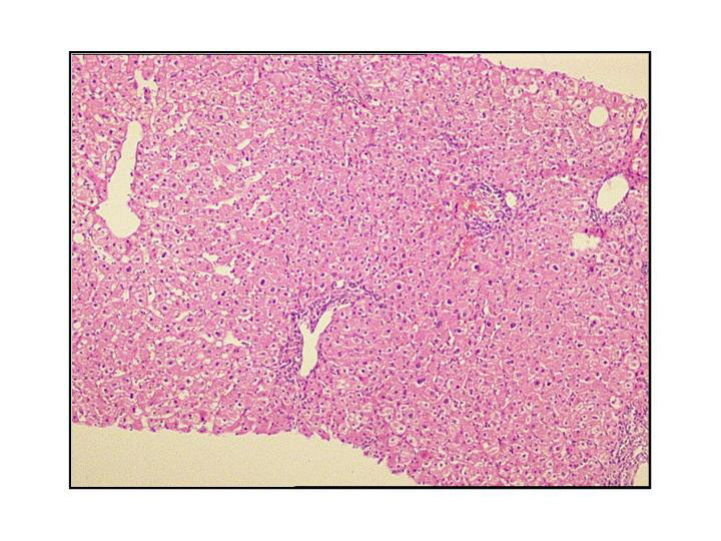
In the second biopsy, inclusions and chronic hepatitic changes are dramatically reduced, (H&E, ×200).

Serum fibrinogen level was within normal limits. We performed tests for qualitative fibrinogen function. Prothrombine time, partial thromboplastin time and thrombin time were also within normal limits. The patient had no history of prolonged bleeding episodes, menorrhagia, or epistaxis. Since infectious, auto-immune or metabolic diseases were excluded, a drug effect was suspected. Therefore, estrogen therapy was ceased and the patient was followed monthly in order to check transaminase levels. Her serum transaminase levels decreased gradually at each monthly visit and normalized at the third month. Then we decided to perform a control liver biopsy. Parenchymal and portal inflammation, steatosis and necroinflammatory activity were remarkably reduced in the second liver biopsy (Figure 4). Again, the same staining procedures were applied. Compared to the first biopsy, fibrinogen positive inclusions decreased dramatically to the point where only one hepatocyte contained an inclusion. Fibrotic activity and nuclear dysplastic findings remained almost the same.

## Conclusion

The patient had elevated transaminase levels without any accompanying clinical symptoms. Her first liver biopsy revealed cytoplasmic inclusion bodies within hepatocytes associated with chronic hepatitis and mild fibrotic activity. The inclusions were sharply bordered and faintly eosinophilic together with a ground-glass appearance. They were negative to PAS and colloidal iron; therefore type IV glycogenoses and Lafora's disease were ruled out on morphological grounds, respectively. No staining with hepatitis antigens and α1-antitrypsin was observed immunohistochemically. Diffuse and strong positivity for fibrinogen was observed. Her serum fibrinogen levels, in addition to prothrombin, partial thromboplastin and thrombin times – excluding dysfibrinogenemia – were within normal limits. The patient was not the offspring of a consanguineous marriage.

Fibrinogen storage in hepatocytes has been previously reported in patients with or without hypofibrinogenemia. The disease was first shown in German families as a familial hypofibrinogenemia [4,5]. Later, specific mutations in hereditary cases were reported [6,7]. Callea et al., placed fibrinogen storage disorders in the ERSD group along with α1-antitrypsin and α1-antichymotrypsin deficiencies, and proposed a classification of fibrinogen storage diseases based on morphologic and clinical evidence, and thus divided the entity into three types [3]. In type I, the inclusions are defined as round or polygonal, weakly eosinophilic cytoplasmic deposits with irregular borders. This type is a hereditary hypofibrinogenemia, genetically characterized by the presence of mutant variants of the fibrinogen molecule, namely fibrinogen Brescia and fibrinogen Aguadilla [3,6,7]. The other two types are rarer. Type II inclusions are large, hyaline bodies that occupy the entire cytoplasm and result in a ground-glass appearance, while round, eosinophilic globules surrounded by a clear halo constitute the inclusions of type III. At the ultrastructural level, type I inclusions are characterized by the presence of densely packed tubular structures, arranged in curved bundles resulting in a finger-print-like appearance. Type II inclusions appear as granular or fragmented fibrillar material and type III inclusions contain a central core resembling the tubular structures of Type I and a surrounding similar to that of Type II. In all types, the inclusions correspond to dilated cisternae of RER.

Cases of fibrinogen storage disease without hypofibrinogenemia have been recently reported. One such patient had atypical type of cytoplasmic inclusions and was considered to demonstrate a congenital case [8]. Two other patients revealed fibrinogen storage in the liver during an acute infectious episode together with Type II inclusions. The latter were thought to have transient, acquired forms of the disease [9].

The inclusions observed in our patient were large, hyaline bodies occupying the entire cytoplasm, resulting in a ground-glass appearance. This morphology is consistent with Type II inclusions. Neither the characteristics of Type I inclusions, such as irregular borders, a clear halo surrounding the inclusion or an acicular shape, nor the findings consistent with Type III inclusions [round, eosinophilic globules with a clear halo around] were present in our patient. Electron microscopy revealed homogenous, granular material within dilated ER, which are also the characteristics of Type II inclusions. Tubular structures found in Type I and partially in Type III inclusions were not present either. These findings allowed us to consider the patient's inclusions to be Type II.

In the first biopsy, fibrinogen storage was accompanied with accumulation of complement 4 and α fetoprotein. This simultaneous deposition of multiple proteins destined for secretion indicates a dysfunction in the secretory apparatus, rather than the retention of a single mutant protein [10]. Callea et al., claimed that this finding may help distinguish between the acquired and congenital forms of the disease [3]. A single, genetically abnormal protein cannot be translocated from RER to smooth endoplasmic reticulum (SER), therefore it accumulates in RER [11]. This type of selective and exclusive retention of fibrinogen defines the congenital and the more frequent form of the disease. On the other hand, structurally normal proteins can get trapped in RER, SER and/or secretory vesicles due to a secretory dysfunction, which might be caused by exogenous agents like alcohol, and by drugs such as colchicine [3]. Thus in our case, a drug-induced, acquired form of fibrinogen storage is further supported by the above findings as well as the dramatic resolution of histopathologic and biochemical parameters after cessation of estrogen administration.

We ascribed the findings of chronic hepatitis and mild fibrotic activity in our patient to fibrinogen storage. Indeed, cases without hypofibrinogenemia, as well as those with hereditary hypofibrinogenemia and those that have a mutational basis, were shown to develop chronic hepatitis, fibrosis or cirrhosis, in the literature [3,4,8,9]. Importantly, low levels of fibrinogen does not necessarily accompany the hepatic storage of fibrinogen, which was the case in our patient. This may also support the secondary, acquired nature of the disease.

Relying on the results explained above, we conclude that, in our patient an oral regimen of estrogen is associated with fibrinogen storage disorder. To our knowledge, this is the first case reported to date to show hepatic fibrinogen storage secondary to estrogen ingestion. The liver biopsies of patients with idiopathic liver enzyme abnormalities should be carefully evaluated for cytoplasmic inclusion bodies and, although rare, fibrinogen deposits.

## Pre-publication history

The pre-publication history for this paper can be accessed here:



## Figures and Tables

**Table 1:** Laboratory features of the patient with exogenous estrogen

**Laboratory parameter**	**Result**	**Normal range**
Aspartate aminotransferase (IU/L)	565	0–40
Alanine aminotransferase (IU/L)	545	0–40
Lactate dehydrogenase (IU/L)	424	254–474
Alkaline phosphatase (IU/L)	354	112–330
gamma-glutamyltranspeptidase (IU/L)	98	0–50
Total bilirubin (mg/dl)	0.5	0.2–1.2
Blood urea nitrogen (mg/dl)	18	2–20
Creatinine (mg/dl)	0.4	0.4–1.0
Triglycerides (mg/dl)	154	29–180
Total cholesterol (mg/dl)	180	128–200
Blood glucose (mg/dl)	85	60–110
Blood ammonium-N (mg/dl)	11	40–85
